# New insights into the mechanisms of electroconvulsive therapy in treatment-resistant depression

**DOI:** 10.3389/fpsyt.2025.1614076

**Published:** 2025-08-14

**Authors:** Ana C. Ruiz, Abdul Haseeb, William Baumgartner, Edison Leung, Giselli Scaini, Joao Quevedo

**Affiliations:** ^1^ Center for Interventional Psychiatry, Faillace Department of Psychiatry and Behavioral Sciences at McGovern Medical School, The University of Texas Health Science Center at Houston (UTHealth), Houston, TX, United States; ^2^ Center of Excellence on Mood Disorders, Faillace Department of Psychiatry and Behavioral Sciences at McGovern Medical School, The University of Texas Health Science Center at Houston (UTHealth), Houston, TX, United States; ^3^ Translational Psychiatry Program, Faillace Department of Psychiatry and Behavioral Sciences at McGovern Medical School, The University of Texas Health Science Center at Houston (UTHealth), Houston, TX, United States; ^4^ Neuroscience Graduate Program, The University of Texas MD Anderson Cancer Center UTHealth Graduate School of Biomedical Sciences, Houston, TX, United States; ^5^ Translational Psychiatry Laboratory, Graduate Program in Health Sciences, University of Southern Santa Catarina (UNESC), Criciúma, SC, Brazil

**Keywords:** electroconvulsive therapy, major depressive disorder, treatment-resistant depression, interventional psychiatry, neuromodulation

## Abstract

Electroconvulsive therapy (ECT) remains one of the most effective interventions for treatment-resistant depression (TRD), particularly in cases involving severe symptomatology, suicidality, or psychotic features. Despite advancements aimed at enhancing the safety and cognitive tolerability of ECT, concerns about cognitive side effects continue to limit its broader acceptance. A deeper understanding of the mechanisms underlying ECT is therefore critical for refining its use and maximizing clinical outcomes. Through a narrative review of recent literature, this paper synthesizes current evidence comparing the efficacy of ECT, ketamine, and repetitive transcranial magnetic stimulation (rTMS) in the treatment of TRD. Then, the review delves into the neurobiological mechanisms through which ECT exerts its therapeutic effects, including modulation of neurotransmitter systems, enhancement of neurogenesis, changes in brain network connectivity, immune response regulation, neurotrophic signaling, and epigenetic alterations. These mechanistic insights may inform the identification of biomarkers predictive of treatment response. Moving forward, future research guided by interaction mechanisms hypotheses could provide more insights into alternative neuromodulation techniques, optimize ECT procedures, and improve patient-specific treatment approaches to enhance therapeutic benefits while minimizing adverse effects.

## Introduction

Treatment-resistant depression (TRD) represents a subtype of major depressive disorder (MDD) characterized by an inadequate response to standard antidepressant treatments. While various definitions and staging models exist, a commonly accepted criterion proposed by the U.S. Food and Drug Administration (FDA) is the failure to achieve a satisfactory response after at least two adequate trials of different antidepressant medications (1). This lack of consensus on a precise definition reflects the complexity of TRD and underscores the need for individualized treatment approaches. TRD remains a significant clinical challenge, affecting approximately 30-40% of individuals with MDD. These patients experience extended periods of illness while struggling with disabling symptoms such as hopelessness, anhedonia, and cognitive dysfunction. Additionally, the chronic nature of their condition increases their risk for a wide range of psychiatric and somatic comorbidities, such as chronic suicidality, anxiety disorders, substance abuse, and cardiovascular disease (2).

Introduced in the 1930s, ECT emerged as a groundbreaking intervention for severe psychiatric conditions. Its application in treating depression, particularly in cases resistant to other treatments, has been well-documented. A study from the 1940s reported that 80% of patients receiving ECT experienced symptomatic improvement, compared to 50% in the control group. Additionally, the average length of hospitalization for the ECT group was significantly shorter, underscoring its efficacy in managing severe depression (3). In modern clinical practice, ECT remains a highly effective treatment for TRD and is considered a first-line treatment for severely depressed patients who require a fast response because of a high suicide or homicide risk, extreme agitation, life-threatening inanition, psychosis, or stupor (4). Beyond its established use for severe TRD, bipolar disorder, schizophrenia, and catatonia, electroconvulsive therapy (ECT) is also being investigated for a broader range of psychiatric and neuropsychiatric conditions. These include post-traumatic stress disorder (PTSD), Parkinson’s disease with psychiatric symptoms, neuropsychiatric complications associated with COVID-19, and perinatal psychiatric disorders, where pharmacologic treatments may pose risks (5). Additionally, ECT is increasingly being applied in acute medical scenarios where a rapid therapeutic response is essential. For medically unstable patients suffering from severe somatic comorbidities, such as dehydration, malnutrition, or profound weight loss, ECT can facilitate urgent clinical stabilization. Emerging evidence also supports its efficacy in managing intractable delirium, particularly in intensive care settings, and in select cases of refractory or super-refractory status epilepticus, where standard treatments have proven ineffective and ECT has been associated with clinical improvement (6, 7).

Although ECT is highly effective in the rapid treatment of various psychiatric disorders and symptoms, it continues to be an underutilized and stigmatized intervention (8), mainly due to its cognitive side effects, such as anterograde and retrograde amnesia. However, ongoing research continues to shed light on its mechanisms of action and develop strategies to mitigate adverse effects, reinforcing its role as a vital option in treating TRD (9). Moreover, recent advancements in ECT techniques have significantly enhanced both its safety and therapeutic efficacy. Innovations in dosing parameters, electrode placement strategies, and the integration of augmenting agents have been meticulously designed to optimize clinical outcomes while minimizing adverse effects. These refinements emphasize the evolving role of ECT in contemporary psychiatric practice, broadening its applicability while ensuring greater precision and safety in treatment delivery.

This review aims to synthesize recent findings on the effectiveness and biological mechanisms of ECT in treating TRD. It includes a discussion of studies that compare ECT with ketamine and repetitive transcranial magnetic stimulation (rTMS), focusing on differences in clinical outcomes. Furthermore, we highlight emerging insights into the neurobiological mechanisms underlying ECT's antidepressant effects, emphasizing pathways implicated in its therapeutic action. By integrating these findings, this review offers a comprehensive overview of the current state of ECT research and outlines promising directions for optimizing its clinical utility in TRD.

## Review methodology

This narrative review was conducted using a targeted literature search conducted across multiple databases, including PubMed, PsycINFO, and Scopus. The strategy utilized combinations of terms such as “electroconvulsive therapy,” “ECT,” treatment-resistant depression,” “mechanisms,” “efficacy,” “neuroplasticity,” “cognitive effects,” “biomarkers,”, “rTMS,” and “ketamine,” In addition to database searches, we performed manual screening of relevant articles from ECT-specific and interventional psychiatry journals, including The Journal of ECT, Brain Stimulation, and Neuropsychopharmacology.

We prioritized peer-reviewed English language publications, including systematic reviews, meta-analyses, randomized controlled trials (RCTs), mechanistic studies, and high-impact narrative reviews. Studies were included if they addressed the clinical efficacy, biological mechanisms, or safety profile of ECT in TRD or related neuropsychiatric conditions.

Exclusion criteria included non-peer-reviewed content, case reports with unclear methodology, or studies focused exclusively on other disorders without relevance to TRD or ECT. This methodology aimed to synthesize foundational and emerging findings while capturing the comparative landscape between ECT, rTMS, and ketamine. The final selection includes over 80 references, representing a balance of clinical and mechanistic perspectives to guide future research and practice.

## Comparative efficacy of ECT

Electroconvulsive therapy (ECT), ketamine, and rTMS are among the most effective treatments for individuals with TRD, each offering distinct clinical advantages (**Table 1**). ECT remains the gold standard, particularly for patients with severe symptoms, suicidality, or psychotic features, or late-life depression, where its efficacy is consistently supported by meta-analyses and geriatric trials. Moreover, older adults often respond more robustly to ECT than younger populations, likely reflecting age-related neurobiological differences and the distinct clinical characteristics of late-life depression (10, 11). However, its broader use is limited by the need for anesthesia and the risk of cognitive side effects, as well as associated costs. Moreover, ECT’s requirement for general anesthesia and muscle relaxation imposes procedural constraints and hospital burdens. In particular, it affects patients with complex medical conditions. Individuals with cardiovascular conditions, such as recent myocardial infarction, unstable coronary artery disease, congestive heart failure, or arrhythmias, as well as those with pulmonary comorbidities (e.g., COPD or OSA). These patients require extensive pre-anesthetic evaluation, which may lead to delays or disqualification from treatment (12). rTMS, on the other hand, offers a non-invasive alternative that targets specific brain regions through magnetic pulses, without the need for anesthesia and with generally good tolerability. Ketamine, an N-methyl-D-aspartate (NMDA) receptor antagonist, has gained recognition for its rapid antidepressant effects, more favorable cognitive profile, and ease of administration. This section examines current evidence comparing ECT, ketamine, and rTMS in the treatment of TRD, focusing on onset of therapeutic effects, cognitive outcomes, and durability of response.

**Table 1:** Comparison of neuromodulation treatments for TRD.

Treatment	Pros	Cons
*Electroconvulsive Therapy (ECT)*	• Most effective for severe TRD, especially with psychotic features or suicidality. • Particularly effective in older adults, with higher response and remission rates in late-life depression • Rapid symptom relief, often within days. • High remission rates compared to other treatments. • Long-standing clinical use with well-established efficacy.	• Cognitive side effects, especially memory loss (more common with bilateral ECT). • Requires anesthesia and muscle relaxants, increasing medical risks. • Stigma and fear surrounding treatment.
*Ketamine*	• Fast-acting antidepressant effects (within hours to days). • Fewer cognitive side effects than ECT. • Non-invasive, no need for anesthesia. • May reduce suicidal ideation quickly, making it useful for crisis intervention. • Can be used in outpatient settings.	• Effects are short-lived, requiring maintenance doses or additional therapy. • High cost and limited insurance coverage. • Risk of dissociation, hallucinations, and blood pressure spikes during administration. • Long-term safety and efficacy remain under investigation. • Potential for misuse or dependence with repeated use.
*Repetitive Transcranial Magnetic Stimulation (rTMS)*	• Non-invasive and generally well-tolerated. • No anesthesia or systemic medications required. • Minimal cognitive side effects. • Suitable for patients who are not candidates for ECT or ketamine.	• Less effective than ECT, particularly in severe TRD. • Slower onset of symptom relief (weeks to months compared to days with ketamine or ECT). • Requires daily sessions over 4–6 weeks. • Variable response rates; not all patients benefit. • Less effective for psychotic or highly severe depression.

A growing body of literature comparing ECT and ketamine for TRD underscores the effectiveness of both interventions. However, they exhibit notable differences in the speed of onset, cognitive effects, and patient outcomes preference. Basso et al. (13), conducted an open-label clinical trial demonstrating that, while both treatments were equally effective, ketamine exerted a more rapid antidepressant effect and improved neurocognitive functions. In contrast, ECT was associated with a mild decline in cognitive performance. In line with this, Ghasemi et al. (14), found that ketamine led to a significantly faster reduction in depressive symptoms within 24 hours compared to ECT. However, its efficacy became comparable to ECT after multiple treatments. A study by Kheirabadi et al. (15), further supported these findings, showing no statistically significant difference in antidepressant efficacy between ECT and ketamine. However, cognitive performance was slightly better in the ketamine group. The KetECT, a multicenter randomized controlled study, added another layer to this evolving comparison, showing that ECT had superior remission rates (62.6% vs. 46.3% for ketamine) (16). The ELEKT-D trial, a multicenter randomized controlled study, found that ketamine was noninferior to ECT in reducing depressive symptoms, with response rates of 55.4% for ketamine and 41.2% for ECT (17). In a *post hoc* secondary analysis, the authors found that patients with the highest severity appeared to benefit more quickly from ECT, potentially due to its robust neurobiological impact. However, ketamine demonstrated higher overall response rates and was especially effective among outpatients with nonpsychotic depression who experienced moderate to severe symptoms of depression (18). A recent meta-analysis encompassing six randomized controlled trials found that both treatments significantly reduced depressive symptoms, with no substantial difference in overall efficacy between the two modalities. Ketamine demonstrated superior memory function improvement compared to ECT. In terms of adverse events, ketamine was associated with significantly higher rates of dissociative symptoms, blurred vision, and dizziness, while demonstrating a lower incidence of muscle pain (19).

Comparative studies have also shown differences in efficacy between rTMS and ECT. In a meta-analysis by Ren et al. (2014), ECT had a higher response rate than high-frequency rTMS (HF-rTMS) for major depression, with response rates of 52.9% for ECT versus 38.3% for HF-rTMS. Similarly, Micallef-Trigona (20) found that ECT was more effective than rTMS, with a significant reduction in HDRS scores in the ECT group. However, rTMS still showed a notable antidepressant effect, suggesting its potential as a viable alternative, especially for patients who may not tolerate ECT. On the other hand, Cano et al. (21), showed that both right unilateral (RUL) ECT versus left dorsolateral prefrontal cortex (lDLPFC) rTMS significantly reduced depressive symptoms in patients with TRD, with QIDS scores decreasing by 30.40% and 36.13%, respectively. Despite higher baseline severity in the ECT group, there was no significant difference in clinical response between the two treatment modalities. A retrospective cohort study showed that ECT exerted a significantly stronger antidepressant effect than rTMS in terms of MADRS-S score reduction, response rate, remission rate, and clinically meaningful change (22). Kaster and Blumberger (23) emphasized the role of rTMS in sequential treatment models, noting that while less effective than ECT, it remains a viable step before ECT for patients seeking non-invasive options. A recent meta-analysis comparing ECT, rTMS, and ketamine in adolescents with TRD confirmed that while ECT remains the most effective, it is often avoided due to stigma and accessibility issues (24). Similarly, a systematic review of RCTs found that ECT is superior to both ketamine and rTMS in overall efficacy. However, ketamine offers faster symptom relief, making it useful in acute interventions (25). Despite these findings, more research is needed to better characterize patient-level predictors of treatment response, which could help guide the selection of optimal treatment modalities based on individual clinical profiles.

ECT, ketamine, and rTMS are three principal interventions for TRD, each characterized by distinct advantages and limitations. ECT has historically been regarded as the most efficacious treatment, particularly for individuals with severe depression, psychotic symptoms, suicidality, or late-life depression. Its well-established effectiveness in older adults should be especially noted, as this population often shows greater clinical response and tolerability (10, 11). However, concerns regarding its associated cognitive impairment, societal stigma, and restricted accessibility have contributed to a decline in its utilization. Ketamine has emerged as a promising alternative due to its rapid antidepressant effects, a more favorable cognitive side effect profile, and its comparatively less invasive administration. rTMS, a non-invasive neuromodulation technique, represents another viable therapeutic option, offering a favorable safety profile but exhibiting more variable efficacy across patient populations (**Table 2**).

**Table 2:** Summary of comparative studies evaluating the antidepressant efficacy of ECT and ketamine or rTMS in TRD.

Study	Design	Sample size	Mainfindings	Limitations
Basso et al. (13),	Naturalistic, non-randomized, comparative study	50 patients	Ketamine and ECT were similarly effective; ketamine acted faster and improved attention and executive function. ECT led to minor cognitive decline.	Non-randomized; Concurrent medication use; No placebo group.
Ghasemi et al. (14),	Randomized, blinded comparison	18 patients	Ketamine showed faster antidepressant effects than ECT within 24h and throughout the second treatment. Similar efficacy by the end (1 week).	Small sample; Titration method was used for ECT; Thiopental as anesthetic (anticonvulsant properties); Short treatment and follow-up period; Did not record seizure durations.
Kheirabadi et al. (15),	Randomized controlled trial	32 patients	No significant difference in HDRS outcomes between ketamine and ECT. Cognitive state was more favorable (not significant) in the ketamine group.	Small sample size; Limited generalizability; No blinding reported.
Ekstrand et al. (16),	Randomized, open-label, non-inferiority trial	186 inpatients	ECT had higher remission rates than ketamine (63% vs 46%, p=0.026). Relapse rates were similar at 12 months. Persistent amnesia was more common with ECT.	No placebo group; Open-label; hospitalized patients only; limited data on long-term cognitive effects.
Anand et al. (17),	Open-label, randomized noninferiority trial	403 randomized (365 treated)	Ketamine was noninferior to ECT in response rates (55.4% vs 41.2%). ECT appeared to be associated with a decrease in memory recall after 3 weeks of treatment, with gradual recovery during follow-up.	Open-label design; Short initial treatment phase; Long-term safety and durability unclear.
Jha et al. (18),	Secondary analysis of an open-label noninferiority randomized clinical trial	365 patients	Ketamine had greater effect in moderately to severely depressed outpatients. ECT was more effective early in very severe or inpatient cases, but effects equalized by week 3.	Secondary analysis; Results not prespecified; Nonblinded; No formal cognitive assessment.
Cano et al. (21),	Prospective, non-randomized observational study.	32 patients	Did not observe a significant difference in clinical response between patients treated with RUL ECT and rTMS (30.40% vs 36.13% change in QIDS score)	Small sample size; Non-randomized; Concomitant medications not controlled; Use of self-report (QIDS) over clinician-rated scales for primary clinical outcomes.
Strandberg et al. (22),	Register-based cohort study	138 patients	ECT was more effective than rTMS (MADRS-S reduction: 15.0 vs 5.6; Response rates: 38% vs 15%). ECT superiority was consistent across age and severity subgroups.	Observational study design; Non-randomized; No formal cognitive assessment.

## Cognitive outcomes

Concerns about cognitive side effects are a significant barrier to the wider acceptance of ECT, despite its effectiveness in treating TRD (26). While early reports highlighted cognitive risks, accumulating evidence indicates that many of these effects are time-limited and, in some cases, reversible. Studies indicate that impairments in attention, executive function, and processing speed typically persist for a brief duration. Most studies find a return to baseline or improvement within weeks to three months after treatment, suggesting that these improvements likely result from both the effects of ECT on the brain and the relief of depressive symptoms (27–29). A large-scale longitudinal study utilizing data from 1,498 patients in the Swedish National Quality Register for ECT found that 25.2% of individuals reported subjective memory worsening six months after treatment. Notably, the strongest predictor of long-term cognitive complaints was residual depressive symptoms, as measured by MADRS-S scores, rather than ECT technical variables such as electrode placement, pulse width, or number of sessions. These findings suggest that patients’ perception of cognitive impairment may reflect unresolved mood symptoms more than the direct neurobiological effects of ECT and highlight the importance of achieving and maintaining full remission (30). Xu et al. (31), demonstrated that ECT is effective in treating young adults with TRD and highlighted the heterogeneous nature of cognitive outcomes during treatment. While global cognition, verbal fluency, and working memory generally remained stable or showed improvement, delayed verbal recall exhibited a transient decline that typically resolved after treatment. Importantly, cognitive impairments were more pronounced among individuals with older age, lower educational attainment, and pre-existing cognitive deficits. A quasi-experimental study examining the timing of autobiographical memory retrieval relative to ECT initiation found that patients who completed the Autobiographical Memory Interview within 24 hours before their first session showed a decline in memory performance post-treatment, whereas those who completed it more than 24 hours in advance demonstrated improvement. These results support the hypothesis that ECT can interfere with the reconsolidation of reactivated memories, a process during which recalled memories become temporarily labile and susceptible to disruption (32).

A recent consensus guideline, developed by a committee of clinical and academic experts from Australia and New Zealand, emphasizes that while most cognitive domains return to baseline or improve shortly after treatment, autobiographical memory loss may endure in a subset of individuals and can be distressing and functionally impairing. Factors influencing cognitive risk include older age, pre-existing brain vulnerability, concurrent lithium use, and extended or bilateral treatment protocols. Although the mean group data suggest recovery, individual-level analyses reveal that some patients may experience significant impairments that are masked by group averages (33).

Collectively, these findings suggest that while most cognitive effects of ECT are transient and may even improve over time, persistent autobiographical memory loss remains a significant concern for a subset of patients. Importantly, perceived cognitive deficits appear to be shaped by both neurobiological and psychological factors, including symptom resolution and timing of memory activation. Future work should focus on refining cognitive monitoring strategies, elucidating individual risk profiles, and exploring behavioral or procedural adjustments to minimize adverse cognitive outcomes without compromising therapeutic efficacy.

## Mechanistic insights

### Neurotransmitter modulation by ECT

The classical monoamine neurotransmitter theory of depression, which posits that a depletion of serotonin, norepinephrine, and dopamine plays a key role in the pathophysiology of the disorder, has historically influenced our understanding of antidepressant mechanisms. Evidence suggests that ECT enhances the neurotransmission of these monoamines.

Post-mortem and *in vivo* imaging studies suggest that ECT increases the availability of serotonin in synaptic clefts by enhancing the function of serotonin transporters (5-HTT) and increasing serotonin receptor sensitivity (34). Hoekstra et al. (35), found an increase in the plasma levels of tryptophan at approximately 24 h post-ECT only in those patients who responded to the treatment, while another study showed total plasma tryptophan levels remained elevated between 2 and 24 hours following ECT, but these alterations were reversible within 48 hours (36). Moreover, studies indicate that serotonin receptor 5-HT_1A_ postsynaptic density is decreased following ECT in depressed patients (37). However, other studies have found that 5-HT_1A_ postsynaptic receptors become more sensitive to serotonin after ECT treatment (38). A significant decrease in brain 5-HT_2_ receptors has also been observed in patients with depression following ECT, mirroring the effects seen with antidepressant medications (38, 39). A pilot study examined how patients undergoing ECT altered the loudness dependency of auditory evoked potentials (LDAEP), a proposed indicator of central serotonergic activity. The results indicated that changes in LDAEP measurements after treatment demonstrated that ECT influences serotonergic activity (40). The differential effects of ECT on various serotonin receptor subtypes highlight the nuanced way in which ECT interacts with the serotonergic system, potentially differing from the more direct actions of selective serotonin reuptake inhibitors (SSRIs) and serotonin-norepinephrine reuptake inhibitors (SNRIs).

ECT has also been shown to influence dopaminergic neurotransmission, which is closely linked to motivation and reward processing. Masuoka et al. (41), showed that ECT can decrease striatal dopamine transporter binding, leading to increased dopamine availability in the synaptic cleft. Preclinical studies using animal models of depression suggest that ECT enhances dopamine release in the nucleus accumbens and striatum, potentially reversing anhedonia, a core symptom of depression (42, 43). Functional imaging studies further support this, demonstrating increased dopaminergic activity in reward-related brain circuits post-ECT (44). These changes might help reduce the psychomotor slowness and anhedonia that are frequently seen in depressed people (45). ​

Beyond monoaminergic changes, ECT significantly impacts the balance between excitatory and inhibitory neurotransmission, particularly through GABAergic and glutamatergic systems. ECT exhibits anticonvulsant properties, leading to a decrease in neural metabolic activity over the course of treatment (46). Repeated seizures induced by ECT result in reduced seizure duration and increased intracortical inhibition, which has been correlated with clinical improvement (47, 48). Bajbouj et al. (47), showed that ECT enhances the activity of inhibitory circuits in the motor cortex, as evidenced by increased intracortical inhibition and cortical silent period duration. Moreover, studies have shown that ECT responders tend to have higher GABA levels at baseline and after a course of ECT when compared to non-responders (49). Moreover, ECT has been found to increase levels of GABA in the anterior cingulate cortex, a region implicated in emotional regulation (49). A proposed neurophysiological theory suggests that mood stability is enhanced by increasing the activity of GABAergic neurons that regulate neurocircuits, attributed to the rise in the seizure threshold caused by the repeated electrically-induced seizures.

The glutamatergic system has also garnered increasing attention for its role in depression and the effects of ECT. Evidence from proton magnetic resonance spectroscopy (¹H-MRS) studies indicates that individuals with MDD exhibit reduced glutamate levels and glutamate/glutamine (Glx) in the anterior cingulate cortex (ACC), a region implicated in mood regulation. Notably, these alterations appear to normalize following successful ECT, correlating with clinical improvement (50–52). Similarly, Njau et al. (53), found that, at baseline, patients had lower Glx levels in the subgenual ACC (sgACC) and higher levels in the left hippocampus compared to healthy controls. After ECT, Glx levels increased in the sgACC and decreased in the hippocampus, with these neurochemical changes correlating with mood improvement. Pfleiderer et al. (54), also observed significantly reduced Glx levels in the left cingulum of depressed patients relative to controls. In patients who responded to ECT, Glx levels normalized and no longer differed from those of healthy individuals, a pattern not observed in non-responders. Supporting these findings, Ermis et al. (55), reported in a longitudinal study that ECT remitters had higher baseline ACC Glx than non-remitters. Notably, after ECT, ACC Glx levels decreased in remitters but increased in non-remitters. Collectively, these studies indicate that severe depression is characterized by regional Glx deficits or dysregulation and support the "anticonvulsant hypothesis" of ECT, which proposes that ECT reverses the GABA/glutamate imbalance underlying the hyperexcitatory state in MDD through glutamate receptor modulation (e.g., NMDA receptors) (56).

It is important to note that these neurotransmitter systems do not function in isolation but rather interact in a complex and interconnected manner. The therapeutic effects of ECT likely arise from a synergistic modulation of these systems, leading to a more balanced neurochemical environment in the brain. The observed changes in neurotransmitter levels and receptor sensitivity following ECT may contribute to the neuroplastic and neuroanatomical changes seen with the treatment, suggesting a cascade of effects that ultimately lead to symptom alleviation.

## Impact of ECT on neurogenesis, brain network connectivity and function

Preclinical and clinical studies indicate that ECT treatment leads to an increase in the count of hippocampal granule cells. Madsen et al. (57), found that ECT induces a more pronounced neurogenic effect compared to traditional pharmacological antidepressants, exhibiting a faster onset of action. Nordanskog et al. (58), showed an increase in hippocampal volumes following ECT using a 3-Tesla MRI scanner. Subsequent longitudinal MRI studies and meta-analyses confirmed increases in hippocampal and amygdala volumes after ECT (59–61).

In a study using longitudinal MRI and neuropsychological testing in two distinct clinical populations (MDD and schizophrenia-spectrum disorders), greater hippocampal volume increases were consistently associated with poorer post-ECT cognitive performance, despite differences in diagnostic profile, electrode placement, and treatment parameters. Notably, the study investigated 42 cortical and subcortical regions and demonstrated that the cognitive outcomes were specifically related to the hippocampus (62). Another study focusing on subjective memory outcomes found that increases in the volume of hippocampal subregions (the right and left dentate gyrus) were associated with greater self-reported memory impairment, particularly in autobiographical recall. Conversely, by the 6-month follow-up, reductions in dentate gyrus volume compared to pre-ECT assessments were observed, and these reductions correlated with improvements in objective cognitive performance (63). These findings highlight a potential paradox: while hippocampal enlargement may reflect a form of treatment-induced neuroplasticity, it may also contribute to cognitive side effects.

Mechanistically, ECT is known to upregulate neurotrophic factors and stimulate neurogenesis within the dentate gyrus, contributing to synaptic remodeling and circuit reorganization. However, the rapid onset and magnitude of observed volume changes suggest that neurogenesis alone cannot fully account for these effects. Corroborating this hypothesis, a study using high-field MRI in mice found that electroconvulsive stimulation induced dose-dependent increases in hippocampal volume. Notably, these volumetric changes persisted even in mice where neurogenesis was ablated through X-ray irradiation, implying that other neuroplastic processes, such as increased synaptic density, contribute to the observed structural alterations (64). While some studies hypothesized that the volumetric changes are a result of the hemodynamic and metabolic shifts that occur during seizures, potentially leading to vasogenic or cytotoxic edema, several studies have found no evidence of increased T2 signal intensity or alterations in diffusivity after ECT, suggesting that edema may not play a central role in the observed structural changes (58, 65, 66). Seizure-induced neuroinflammation remains a plausible contributor, potentially facilitating aberrant neurogenesis and transient blood-brain barrier disruption, which may further explain associated cognitive side effects.

Beyond hippocampal changes, large-scale analyses, such as those from the Global ECT-MRI Research Collaboration (GEMRIC), demonstrated that ECT-induced brain volume changes extend beyond the hippocampus, affecting multiple brain regions, with the most significant changes occurring in the hippocampus and amygdala (67, 68). The magnitude of these volumetric changes was found to be dose-dependent and influenced by the electrical field and induced seizures (69). Moreover, several studies have shown that volume increase was most pronounced in the dentate gyrus, a region associated with neurogenesis, aligning with the neuroplasticity hypothesis (70–72). A recent neuroimaging study by Cano et al. (21),, using structural MRI, found that only ECT caused notable increases in gray matter volume, in the right striatum, pallidum, medial temporal lobe (including the amygdala and hippocampus), anterior insula, anterior midbrain (substantia nigra/ventral tegmental area), and subgenual anterior cingulate cortex. In contrast, no significant structural changes occurred after rTMS, even though both groups showed similar improvements in depressive symptoms. Importantly, these volume changes did not relate to the level of symptom reduction. The findings support the idea that ECT leads to large-scale structural changes in the brain through neuroinflammatory or cellular remodeling processes.

Diffusion-weighted imaging (DWI) and diffusion tensor imaging (DTI) studies provide valuable insights into the microstructural effects of ECT on brain tissue, complementing volumetric findings. In white matter, early reports suggested that ECT enhances fiber integrity, as reflected by increased fractional anisotropy (FA) in regions such as the anterior cingulum and frontal tracts; however, more recent findings have revealed increases in mean diffusivity (MD) and radial diffusivity (RD), which may indicate transient extracellular fluid shifts or blood–brain barrier permeability rather than lasting improvements in white matter organization. Gray matter DWI studies have more consistently reported reductions in MD within the hippocampus and amygdala following ECT, potentially reflecting increased cellular complexity (73). Multisite studies further clarify these effects. Repple et al. (74), found ECT-specific increases in MD in right-hemispheric white matter tracts, with baseline white matter integrity (higher FA, lower MD/RD) predicting greater clinical response. Similarly, Belge et al. (75), reported increases in FA, MD, and axial diffusivity (AD) across several white matter pathways post-ECT, notably in cortico-spinal and fronto-occipital tracts. Although diffusion changes were not directly associated with symptom improvement, both studies suggest that baseline microstructural differences may help identify individuals more likely to benefit from ECT, reinforcing the role of ECT in modulating neural circuits implicated in emotion regulation and neuroplasticity.

Besides these structural modifications, ECT also causes functional changes in the connectivity of the brain network. An overactive default mode network (DMN), especially in the medial prefrontal cortex, is closely associated with ruminative thinking, which is a prevalent disorder associated with depression (76). According to fMRI studies, ECT decreases DMN hyperconnectivity, which is linked to symptom alleviation (77). Additionally, ECT improves stress management and emotional processing by increasing connections between the prefrontal cortex and limbic regions like the hippocampus and amygdala (78). Pang et al. (79), showed that clinical improvement was associated with improved connections within the DMN and between the DMN and the central executive network following ECT. Furthermore, Sun et al. (80), revealed that ECT changed the brain's local and global information-processing processes, and the increase in network metrics was associated with clinical remission. A study using resting-state electroencephalography (RS-EEG) showed that ECT significantly changed the network's topology, indicating a restructuring of functional connections that might be the basis for its antidepressant effects (81). Thus, it is believed that these modifications in functional connectivity contribute to the strong and rapid antidepressant effects of ETC (82, 83).

In summary, ECT causes significant structural and functional changes in the brain, especially in hippocampal circuits, through a complex interplay of neurogenesis, synaptic plasticity, and inflammatory signaling. While these changes contribute to its therapeutic efficacy, they may also explain the temporary cognitive side effects observed in some patients, as they may transiently disrupt pre-existing memory circuits (84). Such effects are commonly observed in the early post-treatment phase, and are typically time-limited, with most patients recovering cognitive function over the weeks to months following treatment (27). These findings underscore the importance of balancing therapeutic efficacy with individualized cognitive risk assessment, particularly in patients with baseline memory vulnerabilities.

### The role of neurotrophic factors

A significant body of evidence indicates that ECT plays a role in upregulating neurotrophic factors. Preclinical and clinical studies have reported that ECT leads to a significant increase in peripheral Brain-Derived Neurotrophic Factor (BDNF) concentrations (85, 86). A meta-analysis indicated that ECT elevates plasma BDNF levels, but not in serum, although this increase was not consistently associated with clinical improvement in depressive symptoms (87). Another study observed that serum BDNF levels increased following ECT, irrespective of its effectiveness, suggesting a direct effect of ECT on BDNF expression (88). ​ More recently, a study showed that BDNF in plasma was significantly lower in TRD patients compared to HCs at baseline but increased following ECT. More importantly, the authors found a potential positive dose-response relationship between doublecortin (DCX) levels in neuron-derived extracellular vesicles (NDEVs) and plasma BDNF, suggesting that neurogenesis and neuroplasticity may be interconnected (89). However, some findings on the changes in BDNF and the response to ECT are controversial, with studies reporting no influence of ECT on serum or plasma BDNF levels during or after ECT series (90–92).

Emerging research also highlights the role of vascular endothelial growth factor (VEGF) in the mechanism of ECT. Pre-clinical studies have shown that ECT increases VEGF levels in the hippocampus region of the brain (93). Clinical studies have also reported increased VEGF levels in the serum and plasma of patients with TRD following ECT (94, 95). Additionally, reduced VEGF levels have been associated with a poor response to ECT, suggesting that this neurotrophin may serve as a predictive biomarker for treatment outcomes (93, 96).

In summary, the increased neurotrophic factor levels following ECT might be associated with the structural and functional brain changes associated with successful treatment. The emerging role of VEGF in the mechanism of ECT, particularly its potential to promote neurogenesis and interact with BDNF, suggests a more complex interplay of neurotrophic factors than initially considered.

### Inflammatory aspects of ECT

ECT has been shown to influence the immune system, with both acute and long-term effects that may contribute to its therapeutic efficacy. Several studies have documented an immediate immune response following ECT, characterized by transient increases in pro-inflammatory cytokines. For example, interleukin-6 (IL-6) and tumor necrosis factor-alpha (TNF-α) levels have been observed to rise shortly after ECT sessions (97). This acute inflammatory response is thought to be part of the body’s physiological reaction to the induced seizure and stress associated with ECT. The elevation in cytokine levels, however, is typically short-lived, returning to baseline within hours to days (98).

While the acute phase of ECT elicits a temporary pro-inflammatory response, long-term effects suggest an overall anti-inflammatory outcome. Chronic inflammation has been linked to depressive disorders, with elevated levels of inflammatory markers such as C-reactive protein (CRP), IL-1β, and TNF-α correlating with symptom severity. Research indicates that repeated ECT sessions contribute to a sustained decrease in inflammatory markers, suggesting an immunoregulatory role (99). Patients who experience symptom relief post-ECT often exhibit reductions in CRP and IL-6 levels, supporting the hypothesis that ECT's antidepressant effects may be partially mediated through immune modulation (100)

Emerging studies suggest that baseline inflammatory marker levels may predict an individual’s response to ECT. Du et al. (101), observed that the ECT group exhibited higher levels of pro-inflammatory biomarkers (IL-1β and IL-6) and lower levels of the anti-inflammatory biomarker (IL-10) at baseline. The authors also found a substantial decrease in IL-1β and IL-6 and an increase in IL-10 levels post-ECT. Moreover, participants who responded to the treatment showed a significant decline in HAMD-17 scores, accentuating ECT's therapeutic potential. Hough et al. (102), found that ECT induces an initial rise in IL-6 and CRP, followed by a post-treatment decline. While these changes did not predict overall depression severity improvements, higher post-treatment IL-6 correlated with better affective and cognitive outcomes, while CRP reductions linked to neurovegetative symptom relief.

In addition to systemic immune responses, ECT has been shown to influence neuroimmune function, particularly through microglial activation. Microglia, the resident immune cells of the brain, play a crucial role in neuroinflammation and neuroplasticity. Studies suggest that ECT may initially activate microglia but later promote an anti-inflammatory state, reducing neuroinflammation associated with psychiatric disorders (103). Studies have also demonstrated that ECT induced the proliferation of NG2-expressing glial cells in the adult rat hippocampus and amygdala (104, 105).

Together, these studies suggest that ECT’s antidepressant effects may involve resetting immune balance, marked by a predictable transition from a transient pro-inflammatory spike to longer-term anti-inflammatory effects. Importantly, the acute increases in inflammatory markers, such as IL-6 and TNF-α, may not be harmful or indicative of adverse outcomes. Rather, they might reflect a normal physiological response to induced seizure activity and may be necessary for initiating downstream neurotrophic and immunoregulatory processes. These early immune shifts are thought to facilitate neuroplasticity and emotional regulation, ultimately contributing to symptom improvement (106). Monitoring both acute and longer-term inflammatory trajectories may offer valuable clinical insights: while short-term elevations are expected and adaptive, sustained reductions in inflammatory tone may underlie durable antidepressant effects. Additionally, tracking post-ECT inflammatory profiles may help identify treatment responders and inform relapse risk, offering a potential avenue for early intervention and individualized care. Despite these promising implications, further research is needed to validate these biomarkers for clinical application and to better understand the mechanistic role of inflammation in mediating ECT outcomes.

### Genetic and epigenetic modifications

Studies have shown that ECT affects DNA methylation patterns, which are essential in neurotrophic signaling, and upregulates genes linked to neuroplasticity and synaptic function. The antidepressant benefits of ECT may be maintained by these molecular changes after the initial post-treatment phase (107). Additionally, a methylome-wide analysis identifies differentially methylated CpG sites annotated in *TNKS* associated with ECT binary response and one differentially methylated CpG site annotated in *FKBP5* associated with continuous response (108). Furthermore (109), found numerous differentially methylated positions and regions (DMPs and DMRs) in genes linked to inflammatory and immune processes, supporting the inflammatory theory of MDD pathogenesis and suggesting a potential role for epigenetic modification in the therapeutic effects of ECT.

A recent study that integrated neuroimaging with transcriptomic gene expression analyses in patients with MDD undergoing ECT revealed a correlation between increased gray matter volume and higher expression levels of MDD risk genes, including CNR1, HTR1A, MAOA, PDE1A, and SST. It also identified ECT-related genes such as BDNF, DRD2, APOE, P2RX7, and TBC1D14 (80). On the other hand, Moschny et al. (110), found no global DNA methylation differences between measured time points (before and after the first and last ECT session) or between ECT responders and non-responders.

These mixed findings highlight the preliminary and heterogeneous nature of epigenetic research in ECT. While some studies point to promising epigenetic signatures associated with treatment response, others report minimal or inconsistent changes, underscoring the need for larger longitudinal and standardized studies to clarify the clinical utility of epigenetic biomarkers in ECT.

### Limitations and future directions

Although mechanistic studies of ECT have advanced our understanding of its biological effects, the current body of evidence is still limited by methodological inconsistencies, underpowered study designs, and substantial patient heterogeneity. These challenges limit the reproducibility and clinical applicability of findings, underscoring the need for more rigorous and standardized research approaches.

Evidence on the impact of ECT on neuroplasticity, inflammation, neurotransmitter systems, and epigenetic regulation remains mixed. Contradictory results across studies, particularly those examining biomarkers such as BDNF, inflammatory cytokines, and methylation signatures, reflect wide variability in sampling techniques, timing of assessments, assay sensitivity, and storage conditions. Similarly, neuroimaging studies frequently differ in modality, analysis pipelines, and regions of interest, contributing to inconsistent reports of hippocampal and network-level changes. These inconsistencies make it difficult to draw firm conclusions and underscore the need for methodological harmonization across studies.

One of the most persistent limitations in the current literature is small sample size. Many mechanistic studies of ECT are conducted with limited cohorts, which reduces statistical power and increases the likelihood of spurious findings. This issue is further exacerbated by heterogeneity in ECT administration protocols, including differences in electrode placement, stimulus intensity, session number, anesthesia protocol, and maintenance strategies. Without standardized treatment and assessment protocols, comparing results across studies or synthesizing them in meta-analyses remains a challenging task.

Patient heterogeneity adds another layer of complexity. TRD encompasses a diverse range of clinical presentations influenced by subtype (e.g., melancholic vs. atypical), age, sex, comorbid medical or psychiatric conditions, medication history, and genetic background. Yet many studies fail to stratify or control for these variables. In particular, the high prevalence of co-occurring disorders such as PTSD and personality disorders can influence treatment response and mechanistic signatures, but are often overlooked in study design. Without accounting for such variation, findings may reflect group averages that obscure clinically meaningful subgroup effects.

Addressing these limitations will require coordinated efforts to standardize research methodology, including ECT protocols, biomarker collection procedures, and cognitive assessments. Multi-site collaborations are needed to increase sample sizes, enhance generalizability, and facilitate replication across diverse populations. Future research should incorporate stratified analyses based on clinical subtypes and comorbidity profiles and move toward integrative, systems-level approaches that combine neuroimaging, molecular, and clinical data. Multi-omics studies will be particularly valuable in identifying converging biological pathways predictive of treatment response. Advanced neuroimaging techniques, including functional MRI and DTI, offer valuable tools for tracking treatment-related brain changes and may aid in identifying biomarkers of response and recovery.

In addition to mechanistic investigations, future work must also prioritize long-term outcomes, particularly in relapse prevention. Although ECT is highly effective acutely, relapse rates remain high, often exceeding 50% within the first-year post-treatment, yet many studies offer limited follow-up. Maintenance strategies involving adjunctive treatments, such as rTMS or ketamine, represent promising avenues for sustaining response and minimizing cognitive burden. For instance, using rTMS as a priming intervention before ECT or ketamine as a post-ECT maintenance therapy may enhance the durability of the effect and mitigate side effects, though these approaches require systematic evaluation. Standardized neuropsychological assessments should also be consistently integrated into these trials to better characterize the cognitive effects of ECT and optimize treatment parameters accordingly. Ultimately, aligning mechanistic research with emerging precision psychiatry models will be essential for tailoring interventions, improving prognosis, and reducing relapse in this complex and high-risk population.

### Conclusion

**Figure 1:**
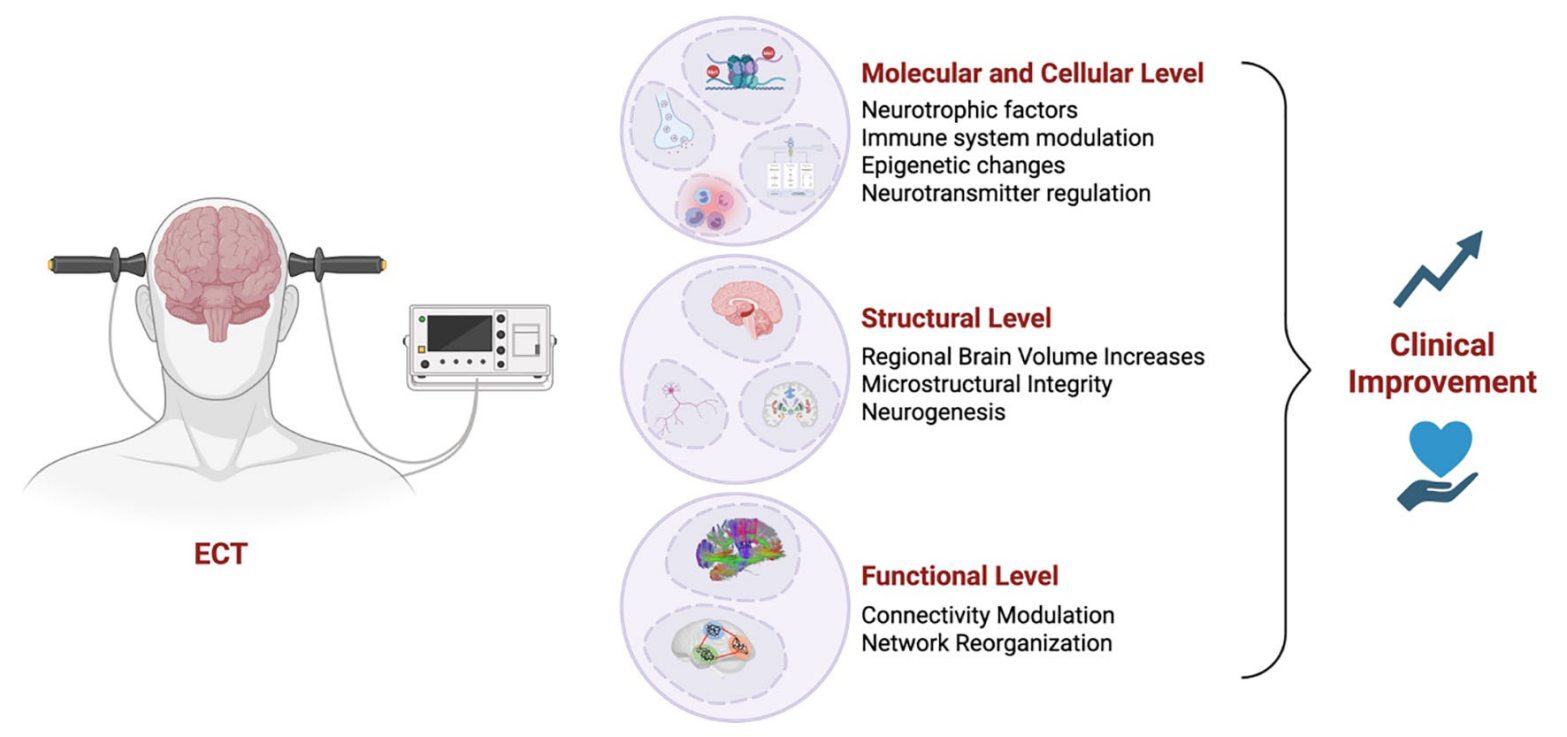
Mechanisms underlying electroconvulsive therapy (ECT)-induced clinical improvement. Schematic illustration summarizing the multilevel mechanisms through which electroconvulsive therapy (ECT) may lead to clinical improvement in individuals with treatment-resistant depression. At the molecular and cellular level, ECT enhances neurotrophic factor expression, modulates immune responses, induces epigenetic modifications, and regulates neurotransmitter systems. At the structural level, ECT has been associated with regional brain volume increases, improved microstructural integrity, and adult neurogenesis, particularly in the hippocampus. Finally, ECT influences functional connectivity and brain network organization. Together, these converging effects contribute to clinical improvement in depressive symptoms.

The findings reviewed in this paper highlight the continued efficacy of ECT in TRD, while shedding light on its mechanistic underpinnings and potential avenues for refinement. Comparative analyses highlight ECT’s superiority in severe cases, particularly when rapid symptom relief is necessary, while alternative treatments, such as ketamine, offer advantages in tolerability and cognitive preservation. Mechanistic insights reveal that ECT may exert its antidepressant effects through the regulation of neurotransmitters, neurogenesis, modulation of brain networks, and neuroimmune modulation, suggesting potential biomarkers for treatment response (**Figure 1**). These insights collectively emphasize the potential of integrating mechanistic understanding with technological advancements, such as fMRI-guided electrode placement and biomarker-driven treatment personalization, to enhance the therapeutic precision of ECT and mitigate its adverse effects. Future research should focus on refining individualized treatment protocols, leveraging neurobiological markers for predicting response, and addressing the stigma surrounding ECT to maximize its accessibility and clinical impact in TRD.

## References

[1] FDA U.S. Food and Drug Administration. Guidance for industry: major depressive disorder: developing drugs for treatment (Fda-2018-0020) (2018). Available online at: https://www.fda.gov/media/113988/download (Accessed Feb 09, 2025).

[2] McIntyre RS Alsuwaidan M Baune BT Berk M Demyttenaere K Goldberg JF. Treatment-resistant depression: definition, prevalence, detection, management, and investigational interventions. World Psychiatry. (2023) 22:394–412. doi: 10.1002/wps.21120, PMID: 37713549 PMC10503923

[3] Tillotson KJ Sulzbach W. A comparative study and evaluation of electric shock therapy in depressive states. Am J Psychiatry. (1945) 101:455–9. doi: 10.1176/ajp.101.4.455

[4] Aetna. Electroconvulsive therapy (Policy number 0445) (2024). Available online at: https://www.aetna.com/cpb/medical/data/400_499/0445.html (Accessed 02/01/2025).

[5] Mukhtar F Regenold W Lisanby SH. Recent advances in electroconvulsive therapy in clinical practice and research. Fac Rev. (2023) 12:13. doi: 10.12703/r/12-13, PMID: 37313441 PMC10259509

[6] Lambrecq V Villega F Marchal C Michel V Guehl D Rotge JY. Refractory status epilepticus: electroconvulsive therapy as a possible therapeutic strategy. Seizure. (2012) 21:661–4. doi: 10.1016/j.seizure.2012.07.010, PMID: 22877995

[7] Nielsen RM Olsen KS Lauritsen AO Boesen HC. Electroconvulsive therapy as a treatment for protracted refractory delirium in the intensive care unit–five cases and a review. J Crit Care. (2014) 29:881 e1–6. doi: 10.1016/j.jcrc.2014.05.012, PMID: 24975569

[8] Maughan D Molodynski A. An international perspective on the acceptability and sustainability of electroconvulsive therapy. BJPsych Int. (2016) 13:10–2. doi: 10.1192/s2056474000000891, PMID: 29093883 PMC5618889

[9] Kritzer MD Peterchev AV Camprodon JA. Electroconvulsive therapy: mechanisms of action, clinical considerations, and future directions. Harv Rev Psychiatry. (2023) 31:101–13. doi: 10.1097/HRP.0000000000000365, PMID: 37171471 PMC10198476

[10] Dominiak M Antosik-Wojcinska AZ Wojnar M Mierzejewski P. Electroconvulsive Therapy and Age: Effectiveness, Safety and Tolerability in the Treatment of Major Depression among Patients under and over 65 Years of Age. Pharm (Basel). (2021) 14(6):582.. doi: 10.3390/ph14060582, PMID: 34207157 PMC8234688

[11] Dong M Zhu XM Zheng W Li XH Ng CH Ungvari GS. Electroconvulsive therapy for older adult patients with major depressive disorder: A systematic review of randomized controlled trials. Psychogeriatrics. (2018) 18:468–75. doi: 10.1111/psyg.12359, PMID: 30073725

[12] Bryson EO Aloysi AS Farber KG Kellner CH. Individualized anesthetic management for patients undergoing electroconvulsive therapy: A review of current practice. Anesth Analg. (2017) 124:1943–56. doi: 10.1213/ANE.0000000000001873, PMID: 28277323

[13] Basso L Bonke L Aust S Gartner M Heuser-Collier I Otte C. Antidepressant and neurocognitive effects of serial ketamine administration versus ect in depressed patients. J Psychiatr Res. (2020) 123:1–8. doi: 10.1016/j.jpsychires.2020.01.002, PMID: 31981856

[14] Ghasemi M Kazemi MH Yoosefi A Ghasemi A Paragomi P Amini H. Rapid antidepressant effects of repeated doses of ketamine compared with electroconvulsive therapy in hospitalized patients with major depressive disorder. Psychiatry Res. (2014) 215:355–61. doi: 10.1016/j.psychres.2013.12.008, PMID: 24374115

[15] Kheirabadi G Vafaie M Kheirabadi D Mirlouhi Z Hajiannasab R. Comparative effect of intravenous ketamine and electroconvulsive therapy in major depression: A randomized controlled trial. Adv BioMed Res. (2019) 8:25. doi: 10.4103/abr.abr_166_18, PMID: 31123668 PMC6477832

[16] Ekstrand J Fattah C Persson M Cheng T Nordanskog P Akeson J. Racemic ketamine as an alternative to electroconvulsive therapy for unipolar depression: A randomized, open-label, non-inferiority trial (Ketect). Int J Neuropsychopharmacol / Off Sci J Collegium Internationale Neuropsychopharmacologicum. (2022) 25:339–49. doi: 10.1093/ijnp/pyab088, PMID: 35020871 PMC9154276

[17] Anand A Mathew SJ Sanacora G Murrough JW Goes FS Altinay M. Ketamine versus ect for nonpsychotic treatment-resistant major depression. New Engl J Med. (2023) 388:2315–25. doi: 10.1056/NEJMoa2302399, PMID: 37224232

[18] Jha MK Wilkinson ST Krishnan K Collins KA Sanacora G Murrough J. Ketamine vs electroconvulsive therapy for treatment-resistant depression: A secondary analysis of a randomized clinical trial. JAMA Netw Open. (2024) 7:e2417786. doi: 10.1001/jamanetworkopen.2024.17786, PMID: 38916891 PMC11200139

[19] Ma Z Wu F Zheng W. Comparative efficacy and safety of ketamine versus electroconvulsive therapy in major depressive disorder: A meta-analysis of randomized controlled trials. Psychiatr Q. (2025). doi: 10.1007/s11126-025-10121-1, PMID: 39964582

[20] Micallef-Trigona B. Comparing the effects of repetitive transcranial magnetic stimulation and electroconvulsive therapy in the treatment of depression: A systematic review and meta-analysis. Depression Res Treat. (2014) 2014:135049. doi: 10.1155/2014/135049, PMID: 25143831 PMC4131106

[21] Cano M Lee E Polanco C Barbour T Ellard KK Andreou B. Brain volumetric correlates of electroconvulsive therapy versus transcranial magnetic stimulation for treatment-resistant depression. J Affect Disord. (2023) 333:140–6. doi: 10.1016/j.jad.2023.03.093, PMID: 37024015 PMC10288116

[22] Strandberg P Nordenskjold A Boden R Ekman CJ Lundberg J Popiolek K. Electroconvulsive therapy versus repetitive transcranial magnetic stimulation in patients with a depressive episode: A register-based study. J ECT. (2024) 40:88–95. doi: 10.1097/YCT.0000000000000971, PMID: 38048154

[23] Kaster TS Blumberger DM. Positioning rtms within a sequential treatment algorithm of depression. Am J Psychiatry. (2024) 181:781–3. doi: 10.1176/appi.ajp.20240604, PMID: 39217438

[24] Faries E Mabe LA Franzen RL Murtaza S Nathani K Ahmed B. Interventional approaches to treatment resistant depression (Dtr) in children and adolescents: A systematic review and meta-analysis. J Affect Disord. (2024) 367:519–29. doi: 10.1016/j.jad.2024.08.212, PMID: 39226935

[25] Saelens J Gramser A Watzal V Zarate CA Lanzenberger R Kraus C. Relative effectiveness of antidepressant treatments in treatment-resistant depression: A systematic review and network meta-analysis of randomized controlled trials. Neuropsychopharmacology. (2024). doi: 10.1038/s41386-024-02044-5, PMID: 39739012 PMC12032262

[26] Kafashan M Lebovitz L Greenspan R Zhao S Kim T Husain M. Investigating the impact of electroconvulsive therapy on brain networks and sleep: an observational study protocol. BMJ Open. (2025) 15:e098859. doi: 10.1136/bmjopen-2025-098859, PMID: 40054874 PMC11891538

[27] Semkovska M Knittle H Leahy J Rasmussen JR. Subjective cognitive complaints and subjective cognition following electroconvulsive therapy for depression: A systematic review and meta-analysis. Aust New Z J Psychiatry. (2023) 57:21–33. doi: 10.1177/00048674221089231, PMID: 35362328

[28] Semkovska M McLoughlin DM. Objective cognitive performance associated with electroconvulsive therapy for depression: A systematic review and meta-analysis. Biol Psychiatry. (2010) 68:568–77. doi: 10.1016/j.biopsych.2010.06.009, PMID: 20673880

[29] Verwijk E Comijs HC Kok RM Spaans HP Tielkes CE Scherder EJ. Short- and long-term neurocognitive functioning after electroconvulsive therapy in depressed elderly: A prospective naturalistic study. Int Psychogeriatr. (2014) 26:315–24. doi: 10.1017/S1041610213001932, PMID: 24280446

[30] Tornhamre E Hammar A Nordanskog P Nordenskjold A. Who Is at Risk of Long-Term Subjective Memory Impairment after Electroconvulsive Therapy? J Affect Disord. (2025) 372:324–32. doi: 10.1016/j.jad.2024.12.028, PMID: 39644929

[31] Xu SX Xie XH Yao L Chen LC Wan Q Chen ZH. Trajectories of efficacy and cognitive function during electroconvulsive therapy course in young adults with treatment-resistant depression. Neuropsychiatr Dis Treat. (2023) 19:267–81. doi: 10.2147/NDT.S394155, PMID: 36744206 PMC9893845

[32] Wiedemann L Trumm S Bajbouj M Grimm S Aust S. The influence of electroconvulsive therapy on reconsolidation of autobiographical memories: A retrospective quasi-experimental study in patients with depression. Int J Clin Health Psychol. (2023) 23:100412. doi: 10.1016/j.ijchp.2023.100412, PMID: 37780809 PMC10534256

[33] Porter RJ Baune BT Morris G Hamilton A Bassett D Boyce P. Cognitive side-effects of electroconvulsive therapy: what are they, how to monitor them and what to tell patients. BJPsych Open. (2020) 6:e40. doi: 10.1192/bjo.2020.17, PMID: 32301408 PMC7191622

[34] Nordanskog P Larsson MR Larsson EM Johanson A. Hippocampal volume in relation to clinical and cognitive outcome after electroconvulsive therapy in depression. Acta psychiatrica Scandinavica. (2014) 129:303–11. doi: 10.1111/acps.12150, PMID: 23745780 PMC4226425

[35] Hoekstra R van den Broek WW Fekkes D Bruijn JA Mulder PG Pepplinkhuizen L. Effect of electroconvulsive therapy on biopterin and large neutral amino acids in severe, medication-resistant depression. Psychiatry Res. (2001) 103:115–23. doi: 10.1016/s0165-1781(01)00282-7, PMID: 11549400

[36] Palmio J Huuhka M Saransaari P Oja SS Peltola J Leinonen E. Changes in plasma amino acids after electroconvulsive therapy of depressed patients. Psychiatry Res. (2005) 137:183–90. doi: 10.1016/j.psychres.2005.07.010, PMID: 16297983

[37] Lanzenberger R Baldinger P Hahn A Ungersboeck J Mitterhauser M Winkler D. Global decrease of serotonin-1a receptor binding after electroconvulsive therapy in major depression measured by pet. Mol Psychiatry. (2013) 18:93–100. doi: 10.1038/mp.2012.93, PMID: 22751491 PMC3526726

[38] Ishihara K Sasa M. Mechanism underlying the therapeutic effects of electroconvulsive therapy (Ect) on depression. Jpn J Pharmacol. (1999) 80:185–9. doi: 10.1254/jjp.80.185, PMID: 10461762

[39] Plein H Berk M. Changes in the platelet intracellular calcium response to serotonin in patients with major depression treated with electroconvulsive therapy: state or trait marker status. Int Clin Psychopharmacol. (2000) 15:93–8. doi: 10.1097/00004850-200015020-00005, PMID: 10759340

[40] Dib M Lewine JD Abbott CC Deng Z-D. Electroconvulsive therapy modulates loudness dependence of auditory evoked potentials: A pilot meg study. Front Psychiatry. (2024) 15:1434434. doi: 10.3389/fpsyt.2024.1434434, PMID: 39188521 PMC11345267

[41] Masuoka T Tateno A Sakayori T Tiger M Kim W Moriya H. Electroconvulsive therapy decreases striatal dopamine transporter binding in patients with depression: A positron emission tomography study with [. Psychiatry Res Neuroimaging. (2020) 301:111086. doi: 10.1016/j.pscychresns.2020.111086, PMID: 32464340

[42] Kobayashi K Imoto Y Yamamoto F Kawasaki M Ueno M Segi-Nishida E. Rapid and lasting enhancement of dopaminergic modulation at the hippocampal mossy fiber synapse by electroconvulsive treatment. J Neurophysiol. (2017) 117:284–9. doi: 10.1152/jn.00740.2016, PMID: 27784811 PMC5225955

[43] Fosse R Read J. Electroconvulsive treatment: hypotheses about mechanisms of action. Front Psychiatry. (2013) 4:94. doi: 10.3389/fpsyt.2013.00094, PMID: 23986724 PMC3753611

[44] Mulders PCR Llera A Beckmann CF Vandenbulcke M Stek M Sienaert P. Structural changes induced by electroconvulsive therapy are associated with clinical outcome. Brain Stimul. (2020) 13:696–704. doi: 10.1016/j.brs.2020.02.020, PMID: 32289700

[45] Landau AM Alstrup AK Audrain H Jakobsen S Simonsen M Møller A. Elevated dopamine D1 receptor availability in striatum of göttingen minipigs after electroconvulsive therapy. J Cereb Blood Flow Metab. (2018) 38:881–7. doi: 10.1177/0271678X17705260, PMID: 28509598 PMC5987930

[46] Deng ZD Robins PL Regenold W Rohde P Dannhauer M Lisanby SH. How electroconvulsive therapy works in the treatment of depression: is it the seizure, the electricity, or both? Neuropsychopharmacology: Off Publ Am Coll Neuropsychopharmacol. (2024) 49:150–62. doi: 10.1038/s41386-023-01677-2, PMID: 37488281 PMC10700353

[47] Bajbouj M Lang UE Niehaus L Hellen FE Heuser I Neu P. Effects of right unilateral electroconvulsive therapy on motor cortical excitability in depressive patients. J Psychiatr Res. (2006) 40:322–7. doi: 10.1016/j.jpsychires.2005.07.002, PMID: 16137698

[48] Brancati GE Medda P Perugi G. The effectiveness of electroconvulsive therapy (Ect) for people with bipolar disorder: is there a specific role? Expert Rev Neurother. (2025) 25:381–8. doi: 10.1080/14737175.2025.2470979, PMID: 40007434

[49] Sanacora G Mason GF Rothman DL Hyder F Ciarcia JJ Ostroff RB. Increased cortical gaba concentrations in depressed patients receiving ect. Am J Psychiatry. (2003) 160:577–9. doi: 10.1176/appi.ajp.160.3.577, PMID: 12611844

[50] Chen X Yang H Cui LB Li X. Neuroimaging study of electroconvulsive therapy for depression. Front Psychiatry. (2023) 14:1170625. doi: 10.3389/fpsyt.2023.1170625, PMID: 37363178 PMC10289201

[51] Moriguchi S Takamiya A Noda Y Horita N Wada M Tsugawa S. Glutamatergic neurometabolite levels in major depressive disorder: A systematic review and meta-analysis of proton magnetic resonance spectroscopy studies. Mol Psychiatry. (2019) 24:952–64. doi: 10.1038/s41380-018-0252-9, PMID: 30315224 PMC6755980

[52] Zhang J Narr KL Woods RP Phillips OR Alger JR Espinoza RT. Glutamate normalization with ect treatment response in major depression. Mol Psychiatry. (2013) 18:268–70. doi: 10.1038/mp.2012.46, PMID: 22565784 PMC3896297

[53] Njau S Joshi SH Espinoza R Leaver AM Vasavada M Marquina A. Neurochemical correlates of rapid treatment response to electroconvulsive therapy in patients with major depression. J Psychiatry neuroscience: JPN. (2017) 42:6–16. doi: 10.1503/jpn.150177, PMID: 27327561 PMC5373714

[54] Pfleiderer B Michael N Erfurth A Ohrmann P Hohmann U Wolgast M. Effective electroconvulsive therapy reverses glutamate/glutamine deficit in the left anterior cingulum of unipolar depressed patients. Psychiatry Res. (2003) 122:185–92. doi: 10.1016/s0925-4927(03)00003-9, PMID: 12694892

[55] Ermis C Aydin B Kucukguclu S Yurt A Renshaw PF Yildiz A. Association between anterior cingulate cortex neurochemical profile and clinical remission after electroconvulsive treatment in major depressive disorder: A longitudinal 1h magnetic resonance spectroscopy study. J ECT. (2021) 37:263–9. doi: 10.1097/YCT.0000000000000766, PMID: 33840802

[56] Lisanby SH McClintock SM Alexopoulos G Bailine SH Bernhardt E Briggs MC. Neurocognitive effects of combined electroconvulsive therapy (Ect) and venlafaxine in geriatric depression: phase 1 of the pride study. Am J Geriatr Psychiatry. (2020) 28:304–16. doi: 10.1016/j.jagp.2019.10.003, PMID: 31706638 PMC7050408

[57] Madsen TM Treschow A Bengzon J Bolwig TG Lindvall O Tingström A. Increased neurogenesis in a model of electroconvulsive therapy. Biol Psychiatry. (2000) 47:1043–9. doi: 10.1016/s0006-3223(00)00228-6, PMID: 10862803

[58] Nordanskog P Dahlstrand U Larsson MR Larsson EM Knutsson L Johanson A. Increase in hippocampal volume after electroconvulsive therapy in patients with depression: A volumetric magnetic resonance imaging study. J ECT. (2010) 26:62–7. doi: 10.1097/YCT.0b013e3181a95da8, PMID: 20190603

[59] Gbyl K Videbech P. Electroconvulsive therapy increases brain volume in major depression: A systematic review and meta-analysis. Acta psychiatrica Scandinavica. (2018) 138:180–95. doi: 10.1111/acps.12884, PMID: 29707778

[60] Gryglewski G Lanzenberger R Silberbauer LR Pacher D Kasper S Rupprecht R. Meta-analysis of brain structural changes after electroconvulsive therapy in depression. Brain Stimul. (2021) 14:927–37. doi: 10.1016/j.brs.2021.05.014, PMID: 34119669

[61] Takamiya A Chung JK Liang KC Graff-Guerrero A Mimura M Kishimoto T. Effect of electroconvulsive therapy on hippocampal and amygdala volumes: systematic review and meta-analysis. Br J psychiatry: J Ment Sci. (2018) 212:19–26. doi: 10.1192/bjp.2017.11, PMID: 29433612

[62] Argyelan M Lencz T Kang S Ali S Masi PJ Moyett E. Ect-induced cognitive side effects are associated with hippocampal enlargement. Transl Psychiatry. (2021) 11:516. doi: 10.1038/s41398-021-01641-y, PMID: 34625534 PMC8501017

[63] Gbyl K Stottrup MM Mitta Raghava J Xue Jie S Videbech P. Hippocampal volume and memory impairment after electroconvulsive therapy in patients with depression. Acta Psychiatr Scand. (2021) 143:238–52. doi: 10.1111/acps.13259, PMID: 33251575

[64] Abe Y Yokoyama K Kato T Yagishita S Tanaka KF Takamiya A. Neurogenesis-independent mechanisms of mri-detecta ble hippocampal volume increase following electroconvulsive stimulation. Neuropsychopharmacology: Off Publ Am Coll Neuropsychopharmacol. (2024) 49:1236–45. doi: 10.1038/s41386-023-01791-1, PMID: 38195908 PMC11224397

[65] Gyger L Ramponi C Mall JF Swierkosz-Lenart K Stoyanov D Lutti A. Temporal trajectory of brain tissue property changes induced by electroconvulsive therapy. Neuroimage. (2021) 232:117895. doi: 10.1016/j.neuroimage.2021.117895, PMID: 33617994

[66] Nuninga JO Mandl RCW Froeling M Siero JCW Somers M Boks MP. Vasogenic edema versus neuroplasticity as neural correlates of hippocampal volume increase following electroconvulsive therapy. Brain Stimul. (2020) 13:1080–6. doi: 10.1016/j.brs.2020.04.017, PMID: 32360430

[67] Oltedal L Bartsch H Sørhaug OJ Kessler U Abbott C Dols A. The global ect-mri research collaboration (Gemric): establishing a multi-site investigation of the neural mechanisms underlying response to electroconvulsive therapy. NeuroImage Clin. (2017) 14:422–32. doi: 10.1016/j.nicl.2017.02.009, PMID: 28275543 PMC5328749

[68] Ousdal OT Argyelan M Narr KL Abbott C Wade B Vandenbulcke M. Brain changes induced by electroconvulsive therapy are broadly distributed. Biol Psychiatry. (2020) 87:451–61. doi: 10.1016/j.biopsych.2019.07.010, PMID: 31561859

[69] Oltedal L Narr KL Abbott C Anand A Argyelan M Bartsch H. Volume of the human hippocampus and clinical response following electroconvulsive therapy. Biol Psychiatry. (2018) 84:574–81. doi: 10.1016/j.biopsych.2018.05.017, PMID: 30006199 PMC6697556

[70] Takamiya A Plitman E Chung JK Chakravarty M Graff-Guerrero A Mimura M. Acute and long-term effects of electroconvulsive therapy on human dentate gyrus. Neuropsychopharmacology: Off Publ Am Coll Neuropsychopharmacol. (2019) 44:1805–11. doi: 10.1038/s41386-019-0312-0, PMID: 30622299 PMC6785137

[71] Nuninga JO Mandl RCW Boks MP Bakker S Somers M Heringa SM. Volume increase in the dentate gyrus after electroconvulsive therapy in depressed patients as measured with 7t. Mol Psychiatry. (2020) 25:1559–68. doi: 10.1038/s41380-019-0392-6, PMID: 30867562

[72] Nuninga JO Mandl RCW Sommer IEC. The dentate gyrus in depression: directions for future research. Mol Psychiatry. (2021) 26:1720–2. doi: 10.1038/s41380-020-0678-8, PMID: 32066830 PMC8440172

[73] Ousdal OT Brancati GE Kessler U Erchinger V Dale AM Abbott C. The neurobiological effects of electroconvulsive therapy studied through magnetic resonance: what have we learned, and where do we go? Biol Psychiatry. (2022) 91:540–9. doi: 10.1016/j.biopsych.2021.05.023, PMID: 34274106 PMC8630079

[74] Repple J Meinert S Bollettini I Grotegerd D Redlich R Zaremba D. Influence of electroconvulsive therapy on white matter structure in a diffusion tensor imaging study. Psychol Med. (2020) 50:849–56. doi: 10.1017/S0033291719000758, PMID: 31010441

[75] Belge JB Mulders PCR Van Diermen L Schrijvers D Sabbe B Sienaert P. White matter changes following electroconvulsive therapy for depression: A multicenter combat harmonization approach. Transl Psychiatry. (2022) 12:517. doi: 10.1038/s41398-022-02284-3, PMID: 36526624 PMC9758171

[76] Stippl A Kirkgöze FN Bajbouj M Grimm S. Differential effects of electroconvulsive therapy in the treatment of major depressive disorder. Neuropsychobiology. (2020) 79:408–16. doi: 10.1159/000505553, PMID: 32344410

[77] Mulders PC van Eijndhoven PF Pluijmen J Schene AH Tendolkar I Beckmann CF. Default mode network coherence in treatment-resistant major depressive disorder during electroconvulsive therapy. J Affect Disord. (2016) 205:130–7. doi: 10.1016/j.jad.2016.06.059, PMID: 27434117

[78] Macoveanu J Craciun S Ketterer-Sykes EB Ysbaek-Nielsen AT Zarp J Kessing LV. Amygdala and hippocampal substructure volumes and their association with improvement in mood symptoms in patients with mood disorders undergoing electroconvulsive therapy. Psychiatry Res Neuroimaging. (2024) 343:111859. doi: 10.1016/j.pscychresns.2024.111859, PMID: 38986265

[79] Pang Y Wei Q Zhao S Li N Li Z Lu F. Enhanced default mode network functional connectivity links with electroconvulsive therapy response in major depressive disorder. J Affect Disord. (2022) 306:47–54. doi: 10.1016/j.jad.2022.03.035, PMID: 35304230

[80] Sun S Yang P Chen H Shao X Ji S Li X. Electroconvulsive therapy-induced changes in functional brain network of major depressive disorder patients: A longitudinal resting-state electroencephalography study. Front Hum Neurosci. (2022) 16:852657. doi: 10.3389/fnhum.2022.852657, PMID: 35664348 PMC9158117

[81] Hill AT Hadas I Zomorrodi R Voineskos D Farzan F Fitzgerald PB. Modulation of functional network properties in major depressive disorder following electroconvulsive therapy (Ect): A resting-state eeg analysis. Sci Rep. (2020) 10:17057. doi: 10.1038/s41598-020-74103-y, PMID: 33051528 PMC7555809

[82] Abbott CC Gallegos P Rediske N Lemke NT Quinn DK. A review of longitudinal electroconvulsive therapy: neuroimaging investigations. J geriatric Psychiatry Neurol. (2014) 27:33–46. doi: 10.1177/0891988713516542, PMID: 24381234 PMC6624835

[83] Sheline YI Price JL Yan Z Mintun MA. Resting-state functional mri in depression unmasks increased connectivity between networks via the dorsal nexus. Proc Natl Acad Sci United States America. (2010) 107:11020–5. doi: 10.1073/pnas.1000446107, PMID: 20534464 PMC2890754

[84] Van der AJ De Jager JE van Dellen E Mandl RCW Somers M Boks MPM. Changes in perfusion, and structure of hippocampal subfields related to cognitive impairment after ect: A pilot study using ultra high field mri. J Affect Disord. (2023) 325:321–8. doi: 10.1016/j.jad.2023.01.016, PMID: 36623568

[85] Angelucci F Aloe L Jiménez-Vasquez P Mathé AA. Electroconvulsive stimuli alter the regional concentrations of nerve growth factor, brain-derived neurotrophic factor, and glial cell line-derived neurotrophic factor in adult rat brain. J ECT. (2002) 18:138–43. doi: 10.1097/00124509-200209000-00005, PMID: 12394532

[86] Altar CA Whitehead RE Chen R Wörtwein G Madsen TM. Effects of electroconvulsive seizures and antidepressant drugs on brain-derived neurotrophic factor protein in rat brain. Biol Psychiatry. (2003) 54:703–9. doi: 10.1016/s0006-3223(03)00073-8, PMID: 14512210

[87] Polyakova M Schroeter ML Elzinga BM Holiga S Schoenknecht P de Kloet ER. Brain-derived neurotrophic factor and antidepressive effect of electroconvulsive therapy: systematic review and meta-analyses of the preclinical and clinical literature. PloS One. (2015) 10:e0141564. doi: 10.1371/journal.pone.0141564, PMID: 26529101 PMC4631320

[88] Luan S Zhou B Wu Q Wan H Li H. Brain-derived neurotrophic factor blood levels after electroconvulsive therapy in patients with major depressive disorder: A systematic review and meta-analysis. Asian J Psychiatr. (2020) 51:101983. doi: 10.1016/j.ajp.2020.101983, PMID: 32146142

[89] Xie XH Xu SX Yao L Chen MM Zhang H Wang C. Altered *in vivo* early neurogenesis traits in patients with depression: evidence from neuron-derived extracellular vesicles and electroconvulsive therapy. Brain Stimul. (2024) 17:19–28. doi: 10.1016/j.brs.2023.12.006, PMID: 38101468

[90] Fernandes B Gama CS Massuda R Torres M Camargo D Kunz M. Serum brain-derived neurotrophic factor (Bdnf) is not associated with response to electroconvulsive therapy (Ect): A pilot study in drug resistant depressed patients. Neurosci Lett. (2009) 453:195–8. doi: 10.1016/j.neulet.2009.02.032, PMID: 19429034

[91] Gedge L Beaudoin A Lazowski L du Toit R Jokic R Milev R. Effects of electroconvulsive therapy and repetitive transcranial magnetic stimulation on serum brain-derived neurotrophic factor levels in patients with depression. Front Psychiatry. (2012) 3:12. doi: 10.3389/fpsyt.2012.00012, PMID: 22375129 PMC3285902

[92] Kleimann A Kotsiari A Sperling W Groschl M Heberlein A Kahl KG. Iv and vi in depressed patients receiving electroconvulsive therapy. J Neural Transm (Vienna). (2015) 122:925–8. doi: 10.1007/s00702-014-1336-6, PMID: 25387785

[93] Maffioletti E Carvalho Silva R Bortolomasi M Baune BT Gennarelli M Minelli A. Molecular biomarkers of electroconvulsive therapy effects and clinical response: understanding the present to shape the future. Brain Sci. (2021) 11(9):1120. doi: 10.3390/brainsci11091120, PMID: 34573142 PMC8471796

[94] Sorri A Jarventausta K Kampman O Lehtimaki K Bjorkqvist M Tuohimaa K. Electroconvulsive therapy increases temporarily plasma vascular endothelial growth factor in patients with major depressive disorder. Brain Behav. (2021) 11:e02001. doi: 10.1002/brb3.2001, PMID: 34342142 PMC8413728

[95] Minelli A Zanardini R Abate M Bortolomasi M Gennarelli M Bocchio-Chiavetto L. Vascular endothelial growth factor (Vegf) serum concentration during electroconvulsive therapy (Ect) in treatment resistant depressed patients. Prog Neuropsychopharmacol Biol Psychiatry. (2011) 35:1322–5. doi: 10.1016/j.pnpbp.2011.04.013, PMID: 21570438

[96] Maffioletti E Gennarelli M Magri C Bocchio-Chiavetto L Bortolomasi M Bonvicini C. Genetic determinants of circulating vegf levels in major depressive disorder and electroconvulsive therapy response. Drug Dev Res. (2020) 81:593–9. doi: 10.1002/ddr.21658, PMID: 32173896

[97] Guloksuz S Arts B Walter S Drukker M Rodriguez L Myint AM. The impact of electroconvulsive therapy on the tryptophan-kynurenine metabolic pathway. Brain Behav Immun. (2015) 48:48–52. doi: 10.1016/j.bbi.2015.02.029, PMID: 25765557

[98] Yrondi A Nemmi F Billoux S Giron A Sporer M Taib S. Significant decrease in hippocampus and amygdala mean diffusivity in treatment-resistant depression patients who respond to electroconvulsive therapy. Front Psychiatry. (2019) 10:694. doi: 10.3389/fpsyt.2019.00694, PMID: 31607967 PMC6761799

[99] Kruse JL Congdon E Olmstead R Njau S Breen EC Narr KL. Inflammation and improvement of depression following electroconvulsive therapy in Treatment-Resistant depression. J Clin Psychiatry. (2018) 79(2):17m11597. doi: 10.4088/JCP.17m11597, PMID: 29489077 PMC6013272

[100] Bioque M Mac-Dowell KS Meseguer A Macau E Valero R Vieta E. Effects of electroconvulsive therapy in the systemic inflammatory balance of patients with severe mental disorder. Psychiatry Clin Neurosci. (2019) 73:628–35. doi: 10.1111/pcn.12906, PMID: 31250493

[101] Du N Wang Y Geng D Chen H Chen F Kuang L. Effects of electroconvulsive therapy on inflammatory markers and depressive symptoms in adolescents with major depressive disorder. Front Psychiatry. (2024) 15:1447839. doi: 10.3389/fpsyt.2024.1447839, PMID: 39524126 PMC11544560

[102] Hough CM Kruse JL Espinoza RT Brooks JO Congdon EJ Norris V. Trajectory of peripheral inflammation during index ect in association with clinical outcomes in treatment-resistant depression. Brain Behav Immun Health. (2025) 43:100925. doi: 10.1016/j.bbih.2024.100925, PMID: 39834556 PMC11743860

[103] Goldfarb S Fainstein N Ben-Hur T. Electroconvulsive stimulation attenuates chronic neuroinflammation. JCI Insight. (2020) 5(17):e137028. doi: 10.1172/jci.insight.137028, PMID: 32780728 PMC7526446

[104] Wennstrom M Hellsten J Ekdahl CT Tingstrom A. Electroconvulsive seizures induce proliferation of ng2-expressing glial cells in adult rat hippocampus. Biol Psychiatry. (2003) 54:1015–24. doi: 10.1016/s0006-3223(03)00693-0, PMID: 14625143

[105] Wennstrom M Hellsten J Tingstrom A. Electroconvulsive seizures induce proliferation of ng2-expressing glial cells in adult rat amygdala. Biol Psychiatry. (2004) 55:464–71. doi: 10.1016/j.biopsych.2003.11.011, PMID: 15023573

[106] Eyre H Baune BT. Neuroplastic changes in depression: A Role for the Immune system. Psychoneuroendocrinology. (2012) 37:1397–416. doi: 10.1016/j.psyneuen.2012.03.019, PMID: 22525700

[107] Tsoukalas I. How does ect work? A new explanatory model and suggestions for non-convulsive applications. Med Hypotheses. (2020) 145:110337. doi: 10.1016/j.mehy.2020.110337, PMID: 33099256

[108] Sirignano L Frank J Kranaster L Witt SH Streit F Zillich L. Methylome-wide change associated with response to electroconvulsive therapy in depressed patients. Trans Psychiatry. (2021) 11:347. doi: 10.1038/s41398-021-01474-9, PMID: 34091594 PMC8179923

[109] Carvalho Silva R Martini P Hohoff C Mattevi S Bortolomasi M Abate M. Unraveling epigenomic signatures and effectiveness of electroconvulsive therapy in treatment-resistant depression patients: A prospective longitudinal study. Clin Epigenet. (2024) 16:93. doi: 10.1186/s13148-024-01704-z, PMID: 39020437 PMC11256624

[110] Moschny N Zindler T Jahn K Dorda M Davenport CF Wiehlmann L. Novel candidate genes for ect response prediction-a pilot study analyzing the DNA methylome of depressed patients receiving electroconvulsive therapy. Clin Epigenet. (2020) 12:114. doi: 10.1186/s13148-020-00891-9, PMID: 32727556 PMC7388224

