# Correction: New insights into the mechanisms of electroconvulsive therapy in treatment-resistant depression

**DOI:** 10.3389/fpsyt.2025.1700480

**Published:** 2025-09-25

**Authors:** Ana C. Ruiz, Abdul Haseeb, William Baumgartner, Edison Leung, Giselli Scaini, Joao Quevedo

**Affiliations:** ^1^ Center for Interventional Psychiatry, Faillace Department of Psychiatry and Behavioral Sciences at McGovern Medical School, The University of Texas Health Science Center at Houston (UTHealth), Houston, TX, United States; ^2^ Center of Excellence on Mood Disorders, Faillace Department of Psychiatry and Behavioral Sciences at McGovern Medical School, The University of Texas Health Science Center at Houston (UTHealth), Houston, TX, United States; ^3^ Translational Psychiatry Program, Faillace Department of Psychiatry and Behavioral Sciences at McGovern Medical School, The University of Texas Health Science Center at Houston (UTHealth), Houston, TX, United States; ^4^ Neuroscience Graduate Program, The University of Texas MD Anderson Cancer Center UTHealth Graduate School of Biomedical Sciences, Houston, TX, United States; ^5^ Translational Psychiatry Laboratory, Graduate Program in Health Sciences, University of Southern Santa Catarina (UNESC), Criciúma, SC, Brazil

**Keywords:** electroconvulsive therapy, major depressive disorder, treatment-resistant depression, interventional psychiatry, neuromodulation

There was a mistake in **Figure 1** as published. Panel is currently labeled “Ischemic Stroke,” but it should correctly read “Functional Level”. The corrected **Figure 1** appears below.

**Figure 1:**
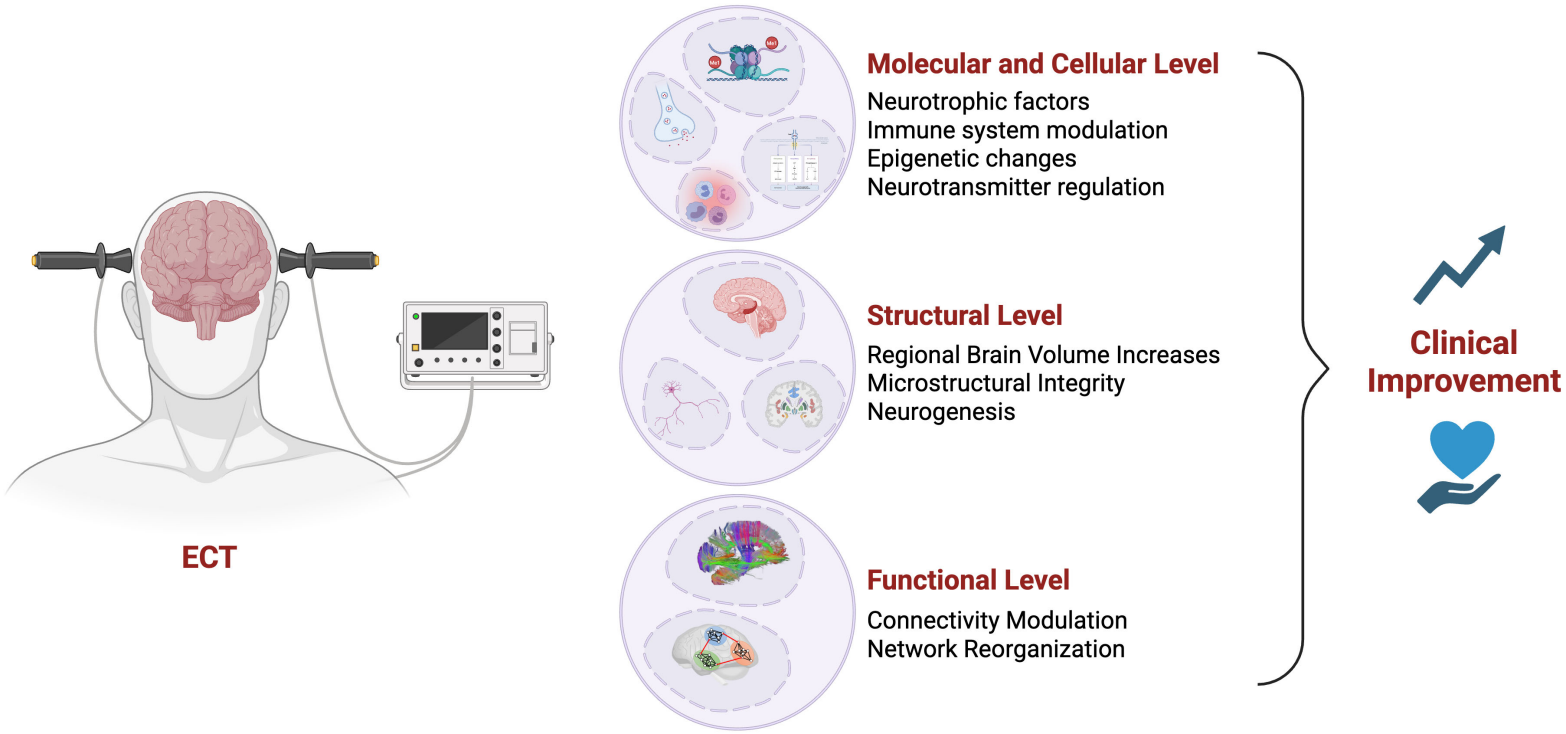
Mechanisms underlying electroconvulsive therapy (ECT)-induced clinical improvement. Schematic illustration summarizing the multilevel mechanisms through which electroconvulsive therapy (ECT) may lead to clinical improvement in individuals with treatment-resistant depression. At the molecular and cellular level, ECT enhances neurotrophic factor expression, modulates immune responses, induces epigenetic modifications, and regulates neurotransmitter systems. At the structural level, ECT has been associated with regional brain volume increases, improved microstructural integrity, and adult neurogenesis, particularly in the hippocampus. Finally, ECT influences functional connectivity and brain network organization. Together, these converging effects contribute to clinical improvement in depressive symptoms.

The original version of this article has been updated.

